# Role of Interleukin- (IL-) 17 in the Pathogenesis and Targeted Therapies in Spondyloarthropathies

**DOI:** 10.1155/2018/2403935

**Published:** 2018-02-12

**Authors:** I-Tsu Chyuan, Ji-Yih Chen

**Affiliations:** ^1^Department of Internal Medicine, Cathay General Hospital, Taipei, Taiwan; ^2^Graduate Institute of Clinical Medicine, College of Medicine, National Taiwan University, Taipei, Taiwan; ^3^Department of Medicine, Division of Allergy, Immunology and Rheumatology, Chang Gung Memorial Hospital, Taoyuan, Taiwan; ^4^College of Medicine, Chang Gung University, Taoyuan, Taiwan

## Abstract

Spondyloarthropathy (SpA) is a unique type of joint inflammation characterized by coexisting erosive bone damage and pathological new bone formation. Previous genetic association studies have demonstrated that several cytokine pathways play a critical role in the pathogenesis of ankylosing spondylitis (AS), psoriatic arthritis (PsA), and other types of SpA. In addition to several well-known proinflammatory cytokines, recent studies suggest that IL-17 plays a pivotal role in the pathogenesis of SpA. Further evidence from human and animal studies have defined that IL-17 and IL-17-producing cells contribute to tissue inflammation, autoimmunity, and host defense, leading to the following pathologic events associated with SpA. Recently, several clinical trials targeting IL-17 pathways demonstrated the positive response of IL-17 blockade in treating AS, indicating a great potential of IL-17-targeting therapy in SpA. In this review article, we have discussed the contributing role of IL-17 and different IL-17-producing cells in the pathogenesis of SpA and provided an outline of therapeutic application of the IL-17 blockade in the treatment of SpA. Other targeted cytokines associated with IL-17 axis in SpA will also be included.

## 1. Introduction

Spondyloarthropathies (SpA) represent a group of chronic inflammatory conditions, involving mainly the axial skeleton (spine and sacroiliac joints), and share a close association with HLA-B27. Ankylosing spondylitis (AS) is the prototypical form of human SpA and characterized in acute and chronic spinal inflammation initiating from sacroiliac joints eventually leading to joint ankylosis [1]. Extensive genome-wide association studies have demonstrated a great number of novel genetic associations beyond HLA-B27 pointing toward inflammatory cytokine pathways in the pathogenesis of AS and other types of human SpA [2, 3], suggesting the role of inflammatory cytokines in the pathogenesis and potential therapeutic application in SpA. Tumor necrosis factor (TNF), interleukin- (IL-) 1, IL-6, and IL-23/IL-17 are major inflammatory cytokine pathways of great interest from serial studies of single nucleotide polymorphisms, cytokine receptors, and associated intracellular signaling molecules. Among these inflammatory cytokines, IL-17 plays a dominant role in the inflammatory and proliferative cascades of human SpA [4, 5]. In this review, we have discussed the contributing role of the IL-17 pathways in the pathophysiology of SpA from current evidence of genetic associations, studies in animal models, and expression of IL-17 in human SpA. Therapeutic effects of the IL-17 blockade in human SpA have also been discussed.

## 2. IL-17 and Its Receptors

The *Il17* gene and IL-17 protein were first discovered from rodent and initially termed as cytotoxic T lymphocyte-associated antigen 8 (CTLA8) [6]. IL-17A is the founding member of the six IL-17 family cytokines (IL-17A to IL-17F) [7–9]. The IL-17 protein consists of 150 amino acids with a molecular weight of 15 kDa, and its gene lies on human chromosome 6p12. Among IL-17 family members, IL-17A and IL-17F are dominant proinflammatory cytokines; they share 55% amino acid resemblance and exist as either disulfide-linked homodimers or heterodimers (IL-17A/F) [9, 10]. Receptors of IL-17 are a heteromeric complex and consist of IL-17RA, IL-17RB, IL-17RC, IL-17RD, and IL-17RE [11, 12]. IL-17 receptors contain conserved structural motifs including an extracellular fibronectin III-like domain and a cytoplasmic SEFIR domain. IL-17A and IL-17F form a homodimer or heterodimer to bind IL-17RA and IL-17RC heterodimeric complexes, thus activating downstream IL-17 receptor intracellular signaling, including nuclear factor-*κ*B (NF-*κ*B), CCAAT/enhancer-binding proteins (C/EBPs) C/EBP*β* and C/EBP*δ*, mitogen-activated protein kinases (MAPKs), and JAK-PI3K and JAK-STAT pathways, to induce antibacterial peptides, proinflammatory chemokines and cytokines, and matrix metalloproteinases (MMPs) for the inflammatory pathogenesis of autoimmune diseases and host defense [13, 14]. IL-17RA is expressed in almost every cell type, including epithelial cells, endothelial cells, fibroblasts, and myeloid cells whilst IL-17RC seems to be a more restricted expression in specific cell types [15].

## 3. Signal Transduction of IL-17

It is now well known that IL-17 can activate the nuclear factor- (NF-) *κ*B pathway [16], and the IL-17A-mediated NF-*κ*B activation is dependent on tumor necrosis factor receptor-associated factor 6 (TRAF6) [17]. Act1, a critical intermediate adaptor between IL-17RA and TRAF6, is recruited after IL-17A stimulation and binds to IL-17RA through SEFIR-dependent interactions [18]. In addition, the TRAF6-binding motif of Act1 further enables it to bind TRAF6 and TGF-*β*-activated kinase 1 to deliver downstream signals, resulting in activation of the canonical NF-*κ*B pathway. Moreover, Act1 can also activate the mitogen-activated protein kinase pathway to stabilize several mRNAs encoding proinflammatory cytokines and chemokines [19]. IL-17-mediated signaling not only promotes proinflammatory cascades but also triggers several regulatory pathways. IL-17A stimulation can recruit TRAF4 to the IL-17 receptor complex, and TRAF4 can be a negative modulator of IL-17-mediated signaling by competing with TRAF6 for Act1 binding [20]. TRAF3 has also been shown to be another negative regulator with a similar action on the IL-17 signaling cascade [21]. Other phosphorylation events, such as phosphorylation of C/EBP*β* by ERK and glycogen synthase kinase 3*β* (GSK3*β*), can also lead to inhibition of IL-17-dependent proinflammatory gene induction [22, 23].

## 4. Sources of IL-17

IL-17A was first cloned from CD4+ T cells, and subsequent evidence suggested the IL-17A-producing CD4+ T cells were distinct from interferon-*γ*- (IFN*γ*-) producing effector T cells [24, 25]. Moreover, these unique subpopulations of CD4+ T cells that produce IL-17 were driven by IL-23 and thus named as Th17 cells [26, 27]. Indeed, these IL-17-producing CD4+ T cells (Th17 cells) were distinct from Th1 and Th2 cells and could develop independently of STAT4 and STAT6, confirming this new T cell subset in the production of IL-17 [28, 29]. Besides Th17 cells, another T cell subset, *γδ* T cells, can respond to IL-23, thus amplifying Th17 responses [30] and producing IL-17 in certain circumstances [31]. In addition to T cells, other IL-17-producing cells include natural killer (NK) cells, mast cells, neutrophils, and innate lymphoid cells; these IL-17-producing cells can produce IL-17 in a specific inflammatory condition possibly through genetic programming [32–35]. Although several animal and human studies suggest these cells produce IL-17 in response to inflammation, the respective contributions of these different cell types to the disease pathology remain to be explored. This raises a critical question for the targeted role of IL-17 in inflammation according to cell-type and disease-type specificity.

## 5. Function of IL-17

The major biological activity of IL-17 is involved in promoting inflammation as earlier studies demonstrated that IL-17 triggers the IL-6 production of synoviocytes from patients with rheumatoid arthritis and this effect is even more enhanced when in synergy with other proinflammatory cytokines (IL-1, TNF) [24, 36, 37]. In addition, IL-17 in chronic inflammation contributes to inhibition of matrix production in chondrocytes and osteoblasts through activating MMPs, resulting in joint destruction and defective tissue repair [38]. IL-17 also increases the expression of receptor activator of NF-*κ*B ligand (RANKL) on osteoblasts and in turn increases RANK signaling in osteoclasts [39–41]. These studies link IL-17 activity to bone destruction, suggesting the potential role of IL-17 in osteoimmunology. In intestinal inflammation, IL-17 also stimulates MMPs, IL6, and IL-8 production from colonic subepithelial myofibroblasts in vitro [42]. In the central nervous system, IL-17 can disrupt the blood-brain barrier tight junction and further facilitates local migration of CD4 T cells, leading to neuroinflammation [43]. All these results support the role of IL-17 in the pathogenesis of tissue-specific and systemic autoimmune diseases.

## 6. IL-17 in AS

Accumulated evidences have demonstrated IL-17 and Th17 effector responses are involved in inflammatory spondyloarthritic condition, including AS, psoriatic arthritis (PsA), reactive arthritis, and undifferentiated spondyloarthritis, suggesting Th17-associated pathways including IL-23R are genetically associated with AS susceptibility [44–46]. A key question to IL-17 in the pathogenesis of SpA is how IL-17 drives inflammation leading to erosive bone damage and pathological new bone formation.

AS is the prototypical form of human SpA and is characterized by joint inflammation, leading to the formation of ectopic new bone and eventually progressive ankylosis of sacroiliac joints. AS has shown a strong genetic association with IL-23R polymorphisms with functional relevance in T cell immune response [44, 47, 48], suggesting genetic variations in the IL-23/IL-17 axis may influence the effector function of Th17 cells in patients with AS. In addition, TNFSF15, TRADD, and CARD9 genes were also reported to be important in the regulation of Th17 cell development and proliferation [49, 50]. Uddin et al. used multiple integrated genomic approaches and identified AS risk alleles that overlap with global immune-related pathways and Th17-related genes [51]. These genetic data results indicate that IL-17 and Th17 effector responses are influenced by both genetic and upstream signal modulation to amplify and perpetuate inflammation.

The importance of IL-17 in the pathogenesis of AS was further defined in several animal and human observational studies. Mice with overexpression of IL-23 represented axial and peripheral enthesitis, and entheseal new bone formation, which is similar to human AS; blockade of the downstream effector cytokine IL-17 significantly reduced disease [52]. IL-17-deficient mice exhibit less joint inflammation in an inflammatory arthritis model [53], and IL-17 receptor-deficient mice also showed decreased proinflammatory cytokine production in the synovium which prevents cartilage destruction [54]. In human AS, IL-17 and IL-23 were elevated in the serum of patients compared to controls and the production IL-17 was even more enhanced when stimulated with IL-23 [55, 56]. Chen et al. also reported similar results of elevated levels of IL-17 and IL-23 in AS patients and further observed that the levels correlate to disease activity measured by Bath Ankylosing Spondylitis Disease Activity Index (BASDAI) scores [57]. These observation studies suggest IL-17 may contribute to the pathogenesis of AS.

Th17 cells are the main cell type in the production of IL-17, and Th17 cells were observed to increase in the blood from patients with AS [58, 59]. Th17 cells specifically expressing KIR3DL2 could respond to the HLA-B27 homodimer and increased in number in AS patients [60]. In addition to Th17 cells, other IL-17-producing T cell subsets have also been identified to be specifically upregulated in AS. For example, increased circulating IL-17-producing IL-23R+ *γδ* T cells were observed in patients with active AS [61]. Furthermore, mast cells were observed to express more IL-17 in SpA and constituted the major IL-17-expressing cell population in the SpA synovium [62]. Increased expression of IL-17 was found predominantly in MPO+ cells and in CD15+ neutrophils in the subchondral bone marrow of inflamed spine from AS patients [4]. All these results suggest that both the innate and adaptive mechanisms drive AS pathogenesis and IL-17-mediated inflammation of AS joints may be tissue- and cell-type specific.

Several antibodies blocking the IL-17/IL-17 receptors have been developed and examined in clinical trials in AS patients over the past few years. At least three monoclonal antibodies have been developed to neutralize IL-17 or block IL-17 receptor signaling: secukinumab (anti-IL-17A), ixekizumab (anti-IL-17A), and brodalumab (anti-IL-17RA). Secukinumab is a fully human monoclonal antibody that selectively binds and neutralizes IL-17. In an earlier phase II clinical trial, secukinumab showed good efficacy in controlling symptoms of AS [63]. Subsequent two large placebo-controlled phase III trials provide further proof of its efficacy in the inhibition of disease activity, similar to what has been seen in previous TNF-blocker trials [64]. Moreover, the drug was superior to placebo both in patients that were naive and in those that had not responded to previous anti-TNF treatment, suggesting the IL-17 pathway inhibits inflammation distinct from that in TNF [65]. Similarly to secukinumab, ixekizumab is another humanized monoclonal antibody that binds and neutralizes IL-17A, and a phase III trial in AS is ongoing (identifier: NCT02696798). Brodalumab is a fully human anti-IL17 RA antibody that inhibits the biological activity of IL-17A, IL-17F, and other IL-17 family members, and a phase III trial in AS is ongoing (identifier: NCT02985983).

Patients with spondyloarthritis exhibit excessive bone formation followed by joint inflammation that can occur at entheses (enthesophytes), near joints on the periosteal surface (osteophytes), or in the spine (syndesmophytes). Whether IL-17 inhibitor treatment might have an effect on the radiographic progression of bone formation and destruction in AS remains to be determined. A recent randomised phase III study on the evaluation of radiographic outcomes of secukinumab in AS shows a low mean progression of spinal radiographic changes over 2 years [66]. However, this secukinumab study did not include a control group. In addition, the effects of blocking IL-17A on periosteal bone formation have not yet been evaluated. Therefore, there is still no firm conclusion about the effects of IL-17 inhibition therapy on radiographic disease progression.

## 7. IL-17 in PsA

PsA belongs to SpA and is characterized by the involvement of the skin (psoriatic plaque), nails, and peripheral and axial joints as well as occasional enthesitis [67, 68]. PsA develops in approximately 25% of psoriasis patients [69], suggesting that the link of psoriasis and PsA may be through a similar immune mechanism and that they share collective pathological events and clinical features. The genetic variations on the IL-23/IL-17 signaling pathway affect the effector function of Th1, and Th17 cells may contribute to the pathogenesis of AS and other SpA [70, 71], and a single nucleotide polymorphism (SNP) in IL-23A and IL-23R has also been shown in association with PsA [72, 73], suggesting that the IL-23/IL-17 pathway may share common genetic background in developing SpA.

Accumulating studies have evidenced the critical role of IL-17 in the pathogenesis of PsA. There were an increased expression of IL-17 observed from psoriatic skin compared to the nonlesioned psoriatic skin, and the upregulation of IL-17 was positively associated with psoriatic disease severity [74, 75]. Fibroblast-like synoviocytes (FLS) from PsA synovium produce more proinflammatory cytokines and MMPs upon IL-17 stimulation [76]. As mentioned earlier, IL-17 can promote osteoclastogenesis resulting in bone erosion through activating the expression of RANKL [77, 78], which can be partially explained by the destructive bone lesions in PsA. All these results suggest IL-17 triggers both skin and joint pathology and inflammation in PsA. The source of IL-17 in psoriatic skin and inflamed joint has been investigated from several observational study results. Th17 cells were observed to have an increased number in both blood and skin lesions from PsA patients, which was also positively correlated with disease activity [79, 80]. In PsA synovium, there were increased IL-17-producing CD4+ effector memory T cell aggregation and expression of functionally active IL-17RA [76]. Other IL-17-producing cells, including mast cells and neutrophils, also represent additional sources of IL-17-mediating psoriasis progression [81]. IL-17-producing mast cells are increased in the synovial fluid of patients with psoriatic arthritis [82] and NKp44+ ILC3 cells are also increased in the peripheral blood and skin in psoriasis [83] and produce IL-17 [84], indicating the IL-17-rich milieu in the inflamed skin and joints of PsA resulting from multiple innate and adaptive IL-17-producing cells. This concept is further confirmed in the murine model of aldara-induced psoriasis, where both skin-invading population of *γδ* T cells and ROR*γ*t CD3-innate lymphocytes which could produce IL-17 contribute to the initiation of psoriasiform plaque formation [85]. Taken together, both IL-17 and IL-17-producing cells contribute to multiple cytokine network and play a pivotal role in the pathogenesis of PsA.

Therapeutic blockade of IL-17 works very well for psoriasis and is relatively good for PsA. In psoriasis, phase III clinical trials for secukinumab have demonstrated that IL-17 inhibition therapy can achieve a 75% improvement of the Psoriasis Area and Severity Index (PASI75) in more than 75% of patients with plaque psoriasis response, and a significant portion of the patients also achieved PASI90 and PASI100 responses [86, 87]. Similar to secukinumab, around 80% to 90% of patients treated with ixekizumab [88, 89] and brodalumab [90, 91] achieved a PASI75 response, indicating that the IL-17 pathway is critical for keratinocytes and infiltrating immune cells in the skin of psoriasis. As for PsA, IL-17 inhibition therapy seems to have a modest effect on the inhibition of joint inflammation and bone destruction. In a phase II clinical trial for secukinumab in patients with moderate-to-severe PsA, a trend towards improvement was demonstrated in the secukinumab group although it did not meet statistical significance [92]; a subsequent phase III clinical trial is ongoing to verify its efficacy in PsA (identifier: NCT02771210). Although a phase III clinical trial for brodalumab in PsA was halted initially because of increased suicidal ideation and suicide events in previous psoriasis clinical trials [90], a phase III trial for brodalumab in axial SpA is ongoing (identifier: NCT02985983).

## 8. Other Targeted Cytokines Associated with IL-17 Axis in SpA

Previous studies have shown that IL-23 can trigger and drive Th17 responses by expansion of Th17 cells and thus an increase in IL-17 levels. Ustekinumab, a monoclonal antibody against the p40 subunit of IL-12 and IL-23, has shown to be beneficial in the treatment of AS, with improvement of clinical disease score, patient-reported outcome parameters, and MRI score [93], suggesting IL-23/IL-17-targeted therapies are very promising for SpA.

IL-17 is the main cytokine of Th17 cells; nevertheless, Th17 cells can also produce some other cytokines including IL-22 [94, 95]. IL-22 belongs to the IL-10 family and is released by several types of CD4+ and CD8+ T cells as well as natural killer T cells, *γδ* T cells, and type 3 ILCs [96]. The IL-17 and IL-22 expression is associated with many autoimmune processes, such as psoriasis and inflammatory bowel disease [97, 98]. In an SpA animal model, although the development of enthesitis and entheseal new bone formation are largely influenced by IL-17/IL-23 axis, enthesitis is specifically dependent on IL-22 [99], and the IL-22-producing entheseal resident cells have shown to activate osteoblast-mediated bone remodeling [52], suggesting IL-22 may coordinate with the IL-17/IL-23 pathway in inflammatory bone remodeling. Furthermore, synovial fluid of PsA patients have shown higher concentration of IL-22, and exogenous administration of IL-22 can induce marked proliferation of PsA-derived fibroblast-like synoviocytes [100]. A recent study has further demonstrated that IL-22 increases the proliferation and migration of the human mesenchymal stem cell (MSC) in inflammatory environments, and the MSC osteogenesis is enhanced following IL-22 exposure [101]. All these results suggest the potential role of IL-22 in addition to the IL-17/IL-23 axis in bone remodeling and provide a novel pathway for exploring pathological and inflammatory osteogenesis in SpA.

## 9. Conclusions

In summary, accumulated evidence supports the critical role of IL-17 and IL-17-producing cells in the joint inflammation, cartilage damage, and bone remodeling associated with SpA. Animal models as well as human genetic and observation studies provided strong mechanistic rationales for targeting IL-17-associated pathways in the treatment of SpA. Several clinical trials showed that blockade of IL-17 successfully restores joint inflammation and remodeling in SpA, providing effective and well-tolerated alternatives to conventional treatment in SpA.
